# Antimicrobial effect of chlorhexidine on *Aggregatibacter actinomycetemcomitans * biofilms associated with peri-implantitis

**DOI:** 10.15171/joddd.2016.028

**Published:** 2016-08-17

**Authors:** Zeinab Kadkhoda, Zeinab Amarlu, Saeed Eshraghi, Nazanin Samiei

**Affiliations:** ^1^Associate Professor, Department of Periodontics, School of Dentistry, Tehran University of Medical Sciences, Tehran, Iran; ^2^Dentist, Birjand, Iran; ^3^Associate Professor, Department of Pathobiology, School of Public Health, Tehran University of Medical Sciences, Tehran, Iran; ^4^Postgraduate Student, Department of Periodontics, School of Dentistry, Tehran University of Medical Sciences, Tehran, Iran

**Keywords:** Aggregatibacter actinomycetemcomitans, chlorhexidine, peri-implantitis

## Abstract

***Background.*** This study aimed to assessthe antimicrobial effect of chlorhexidine (CHX) on *Aggregatibacter actinomycetemcomitans* biofilms isolated from subgingival plaque of peri-implantitis lesions.

***Methods.*** Thirteen patients requiring peri-implantitis treatment were consecutively selected and their subgingival biofilm was collected by inserting fine sterile paper points into peri-implant pockets for 15 seconds. *A. actinomycetemcomitans* was isolated from the subgingival biofilm and cultured. In this study, the standard strain of *A. actinomycetemcomitans* served as the positive control group and a blank disc impregnated with water served as the negative control; 0.1 mL of the bacterial suspension was cultured on specific culture medium and blank discs (6 mm in diameter) impregnated with 0.2%CHX mouthrinse (Behsa Pharmaceutical Co.) and negative control discs were placed on two sides of the bacterial culture plate. The size of growth inhibition zone was measured by a blinded independent observer in millimetres.

***Results.*** According to the results of disc diffusion test, the mean diameter of growth inhibition zone of *A. actinomycetemcomitans* around discs impregnated with CHX was larger in both standard (positive control) and biofilm samples of A. actinomycetemcomitans compared to the negative control group (blank disc) (P<0.001).

***Conclusion***. Use of0.2% CHX mouthwash had antibacterial effects on *A. actinomycetemcomitans* species isolated from peri-implantitis sites.

## Introduction

Peri-implantitis is defined as a destructive inflammatory process of the peri-implant soft and hard tissues, often resulting in loss of supporting bone structure beyond biological bone remodelling.^1,2^ Reports are variable regarding the incidence and prevalence of peri-implantitis and a prevalence of 6.61% over a 9‒14-year period,^3^ 23% during a 10-year period^4^ and 36.6% during an average of 8.4 years of loading.^5^ The specific role of bacteria in peri-implantitis was argued recently. Periodontal pathogens such as *A. actinomycetemcomitans*, *Porphyromonas gingivalis*, *Porphyromonas intermedia*, *Tannerella forsythia* and *Treponema denticola* have been isolated from peri-implantitis pockets.^6^At present, association of no single microorganism has been confirmed with implant failure but a shift has been noticed from a predominately gram-positive non-motile, aerobic and facultative anaerobic microorganisms towards gram-negative, motile, anaerobic bacteria.^7^

The available clinical protocols for prevention and treatment of peri-implantitis are variable and include non-surgical and surgical approaches but because of incomplete removal of biofilm due to difficult accessibility, screw-shaped design of the implant and the rough implant surface, the predictability of non-surgical treatment, particularly mechanical debridement, has been questioned^8,9^and additional treatments such as laser therapy, use of antibiotics and/or antiseptics such as chlorhexidine, stannous fluoride, hydrogen peroxide and 35% phosphoric acid gel have been suggested recently.^10^

Chlorhexidine (CHX) has been the gold standard oral antiseptic for plaque control for the past 2 decades with no possibility of systemic toxicity, microbial infection or supra-infection.^11^ A recent review of the literature also revealed that rinsing with CHX and saline solution was efficient to decontaminate implants with sandblasted/acid-etched and titanium plasma-sprayed surfaces.^10^

Considering the predominant role of CHX as the leading oral antiseptic in surgical and non-surgical treatment of peri-implantitis and gap of published information on the susceptibility of *A. actinomycetemcomitans* biofilm to oral antiseptics and mouthwashes, this study sought to assess the antimicrobial effect of CHX on *A. actinomycetemcomitans* biofilm isolated from subgingival plaque of peri-implantitis lesions.

## Methods

The study protocol was approved by the Ethics Committee of Tehran University of Medical Sciences number 4827and written informed consent was obtained from the participants.

### Patient selection

**Figure 1.**
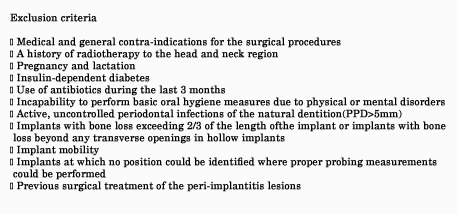
Inclusion criteria.

**Figure 2.**
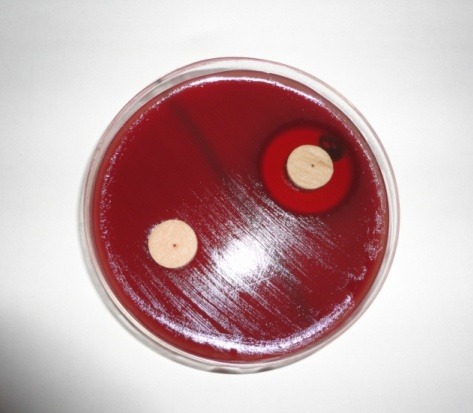
Exclusion criteria.

The participants were selected among patients seeking peri-implantitis treatment in the Department of Periodontics, Tehran University of Medical Sciences. The inclusion and exclusion criteria are presented in Figures 1 and 2. Presence of peri-implant mucositisis often characterized by bleeding on probing and/or suppuration and is usually associated with probing depths >4 mm with any degree of detectable bone loss following initial bone remodelling after implant placement‏.

### Biofilm extraction

The diseased sites were isolated with cotton rolls and subgingival biofilms were obtained using fine sterile paper points (#30) by placing them in peri-implant pockets for 15 seconds. The paper points were immediately placed in a small glass containing thioglycollate broth (Fluid Thioglycollate Medium) and transferred to a laboratory within 30 minutes.

### Transferring the samples

In this study, 1 mL of thioglycollate was used as the transfer medium and immersed in boiling water bath for 10 minutes before sampling. Therefore, oxygen was removed from the medium. The bacteria were separated from the paper points by vortexing for 30 seconds. The samples were then cultured in specific *A. actinomycetemcomitans* culture medium (Brucella agar enriched with lysed sheep blood, vitamin K1, fetal bovine serum, bacitracin, vancomycin and hemin) by a standard loop and incubated in anaerobic conditions in anaerobic jars containing pack-gas and catalyst (which produced CO_2_) at 37°C for 48 hours‏.

After colony growth, star-shaped colonies were sampled. The presence of gram-negative *A. actinomycetemcomitans* was detected by gram staining and microscopic study. In addition, to identify *A. actinomycetemcomitans* biochemical diagnostic tests (oxidase, catalase and glucose tests) were performed onthesamples.

Finally, samples which were assessed in this procedure were frozen and stored at -70°C.

In order to evaluate the effect of CHX on bacteria, frozen *A. actinomycetemcomitans* was placed at room temperature to thaw. Then, 0.1 mL volume of bacterial suspension was diluted in broth to an optical density of 0.5 McFarland concentration. Finally, 50 μL of this suspension was added to specific *A. actinomycetemcomitans* medium (as mentioned before) and incubated at 37°C in the presence of 5% CO_2_ for 24 hours.

### Disc diffusion method

**Figure 3.**
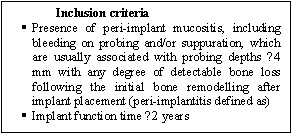
Zone of growth inhibition.

After 24 hours of broth culture (0.5 McFarland), *A. actinomycetemcomitans* bacteria were aseptically subcultured and evenly spread on blood agar plates using a sterile swab. Three to five minutes were allowed for the culture medium to adhere to the plate. Next, blank discs (6 mm in diameter) impregnated with 0.2% CHX mouthrinse (Behsa Pharmaceutical Co.) and negative control discs (not impregnated with0.2% CHX) were placed on the bacterial culture plate using sterile forceps, and were finally incubated in an anaerobic jar at 37°C for 48 hours. The growth inhibition zone was measured as the distance from the edge of the disc to the edge of bacterial colonies by a blinded independent observer in millimetres (Figure 3).

In this study, we used two control groups: one was the standard strain of *A. actinomycetemcomitans* as the positive control group toassess the efficacy of CHX disc and the other one was a blank disc impregnated with water, free of any active agent against *A. actinomycetemcomitans* as the negative control group.

In order to find differences between the two groups, the diameters of growth inhibition zones were analysed by Student’s t-test**.** Statistical significance was set at P < 0.05. SPSS19 (SPSS Inc., Chicago, IL, USA) was used for data analysis.

## Results

A total of 13 patients (4 males and 9 females) with a mean age of 43.85 years and an average probing depth of 6.85 mm were evaluated as shown in Table 1.

**Table 1:** Patients’ age and sex distribution, location and probing depth in peri-implantitis group

**No. of patients**	**Age**	**Sex**		**Location**		**Probing depth**
		**M**	**F**	**Upper**	**Lower**	
13	43.85±1.12	4	9	6	7	6.85±1.35 mm

Six samples were collected from the maxilla (46.2%) and seven from the mandible (53.8%).

According to the results of disc diffusion test (Table 2), the mean diameter of *A. actinomycetemcomitans* growth inhibition zone around discs impregnated with CHX was significantly larger in both standard (positive control) and biofilm samples of *A. actinomycetemcomitans* compared to the negative control group(blank disc), and was 0 after 48 hours of incubation (P<0.001). Negative controls did not demonstrate any zone of growth inhibition.

**Table 2:** The diameter of zone of inhibition in discs impregnated with CHX and negative control disc

**Groups**	**No. of discs**	**Zone of inhibition (mm)**			
		**Max**	**Min**	**Average**	**Standard Deviation**
**CHX Impregnated discs**	13	21	17.8	19.43±1.15	1.15653
**Standard bacterium discs**	4	30	30	30±1.15	1.15653
**Negative control discs**	4	0	0	0	0

Comparison of the mean diameter of *A. actinomycetemcomitans* growth inhibition zone around discs impregnated with CHX in standard groups (in 4 groups) and those obtained from patients with peri-implantitis revealed a significant difference (P < 0.001).

## Discussion

Although data on the susceptibility of *A. actinomycetemcomitans* and oral biofilm to oral antiseptics are scarce, some papers have reported that the use of antibiotics and/or antiseptics such as CHX, stannous fluoride, hydrogen peroxide and 35% phosphoric acid gel along with mechanical and nonsurgical treatments was effective for the treatment of peri-implantitis lesions.^10^

The results of this study indicated that CHX had asignificant effect on biofilms collected from subgingival plaque of patients with peri-implantitis in comparison with no use of CHX. These findings are consistent with those of previous studies.^12,13^

Chlorhexidine with its broad-spectrum gram-positive and gram-negative antibacterial activity is known as the gold standard of oral antiseptics.^14^ Literature review revealed no similar studies on the primary antibacterial effect of oral antiseptics on *A. actinomycetemcomitans* biofilm associated with peri-implantitis. However, CHX proved to have a predictable efficacy in decreasing the bacterial load on titanium surfaces among other antiseptics such as sodium hypochlorite, hydrogen peroxide, essential oils and citric acid, and this may suggest optimal efficacy against peri-implantitis.^15^The mechanism of action of CHX in eliminating the bacteria is related to cationic molecules attached to negatively charged bacterial cell surface and consequent leakage and destruction of the cell wall.^16,17^ Since CHX has no selective ability for destroying bacterial and nonbacterial proteins, mechanical cleaning of implant surface prior to applying CHX is recommended.^18,19^ One of the main advantages of CHX is its prolonged activity but this effect may induce negative side effects such as potential cytotoxicity against cell lines; thus to minimize the possibility of side effects of CHX, rinsing the implant surface would be beneficial to prevent deleterious effects on implant surfaces.^20^ A recent review of the literature also revealed that rinsing with CHX and saline solution was suitable to decontaminate implants with sandblasted/acid-etched and titanium plasma-sprayed surfaces.^10^

Finally, considering the limitations of this in vitro study it can be concluded that CHX has significant effects on *A. actinomycetemcomitans* biofilm extracted from subgingival plaque of patients with peri-implantit is lesions. Although the results of this study are relatively encouraging, further in vitro, ex vivo and animal studies as well as randomized clinical trials are needed to introduce the optimal protocol of using CHX in the treatment of peri-implantitis and achieve greater suppression of anaerobic bacteria on the implant surfaces.

## Conclusion

Within the limitations of this study, the results showed that 0.2% CHX mouthwash had significant antibacterial effects on *A. actinomycetemcomitans* species isolated from subgingival plaque of peri-implantitis patients.

## Acknowledgments

This research was performed in the Department of Periodontics, Faculty of Dentistry and Department of Pathobiology, Faculty of Health Science, Tehran University of Medical Sciences.

## Authors’ contributions

The study concept was developed by ZK and SE, who also contributed to the study proposal. ZA carried out the laboratory procedures. NS drafted the manuscript. ZK and NS had contributions in critically revising the manuscript. All the authors have read and approved the final manuscript.

## Funding

This research was supported by a grant from Tehran University of Medical Sciences & Health Services (grant 89-02-69-10052).

## Competing interests

The authors declare no competing interests with regards to the authorship and/or publication of this article.

## Ethics approval

The study protocol was approved by the Ethics Committee of Tehran University of Medical Sciences number 4827and written informed consent was obtained from all the participants.

## References

[1] Mombelli A (2002). Microbiology and antimicrobial therapy of peri-implantitis. Periodontol 2000.

[2] Rosen P, Clem D, Cochran D, Froum S, McAllister B, Renvert S (2013). Peri-implant mucositis and peri-implantitis: a current understanding of their diagnoses and clinical implications. J Periodontol.

[3] Roos-Jansåker AM LC, Renvert H, Renvert S (2006). Nine- to fourteen-year follow-up of implant treatmentPart II: Presence of peri-implant lesions. J Clin Periodontol.

[4] Marrone A, Lasserre J, Bercy P, Brecx M (2013). Prevalence and risk factors for peri-implant disease in Belgian adults. Clin Oral Implants Res.

[5] Koldsland OC SA, Aass AM (2010). Prevalence of peri-implantitis related to severity of the disease with different degrees of bone loss. J Periodontol.

[6] Hultin M, Gustafsson A, Hallström H, Johansson LÅ, Ekfeldt A, Klinge B (2002). Microbiological findings and host response in patients with peri-implantitis. Clin Oral Implants Res.

[7] Pye A, Lockhart D, Dawson M, Murray C, Smith A (2009). A review of dental implants and infection. J Hosp Infect.

[8] Esposito M, Grusovin M, Worthington H (2012). Interventions for replacing missing teeth: treatment of peri-implantitis. The Cochrane database of systematic reviews.

[9] Mustafa K, Wroblewski J, Hultenby K, Lopez B, Arvidson K (2000). Effects of titanium surfaces blasted with TiO2 particles on the initial attachment of cells derived from human mandibular boneA scanning electron microscopic and histomorphometric analysis. Clin Oral Implants Res.

[10] Subramani K, Wismeijer D (2012). Decontamination of titanium implant surface and re-osseointegration to treat peri-implantitis: a literature review. Int J Oral Maxillofac Implants.

[11] Van Strydonck D, Timmerman M, van der Velden U, van der Weijden G (2005). Plaque inhibition of two commercially available chlorhexidine mouthrinses. J Clin Periodontol.

[12] More G, Tshikalange TE, Lall N, Botha F, Meyer JJM (2008). Antimicrobial activity of medicinal plants against oral microorganisms. J Ethnopharmacol.

[13] Thrower Y, Pinney R, Wilson M (1997). Susceptibilities of Actinobacillus actinomycetemcomitans biofilms to oral antiseptics. J Med Microbiol.

[14] Mathur S, Mathur T, Srivastava R, Khatri R (2011). Chlorhexidine: the gold standard in chemical plaque control. Natl J Physiol Pharm Pharmacol.

[15] Gosau M, Hahnel S, Schwarz F, Gerlach T, Reichert T, Bürgers R (2010). Effect of six different peri-implantitis disinfection methods on in vivo human oral biofilm. Clin Oral Implants Res.

[16] Russell A (1986). Chlorhexidine: antibacterial action and bacterial resistance. Infection.

[17] Rölla G, Melsen B (1975). On the mechanism of the plaque inhibition by chlorhexidine. J Dent Res.

[18] Jones CG (1997). Chlorhexidine: is it still the gold standard?. Periodontol.

[19] de Waal Y, Raghoebar G, Huddleston SJ, Meijer H, Winkel E, van Winkelhoff A (2013). Implant decontamination during surgical peri-implantitis treatment: a randomized, double-blind, placebo-controlled trial. J Clin Periodontol.

[20] Giannelli M, Chellini F, Margheri M, Tonelli P, Tani A (2008). Effect of chlorhexidine digluconate on different cell types: a molecular and ultrastructural investigation. an international journal published in association with BIBRA.

